# Surface Electromyographic Assessment of the Cranio-Cervico-Mandibular System in Healthy Individuals with Different Head Posture Profiles During Portable Electronic Device Use

**DOI:** 10.3390/healthcare14182960

**Published:** 2026-09-10

**Authors:** Marian Turbatu, Alessandro Nota, Teresa Laborante, Laura Pittari, Giulia Deodato, Chiara Maria Galati, Doina Lucia Ghergic, Simona Tecco

**Affiliations:** 1 Dental School, Vita-Salute San Raffaele University, IRCCS San Raffaele Hospital, 20132 Milan, Italy; dr.t.marian@gmail.com (M.T.); teresa.laborante5@gmail.com (T.L.); l.pittari1@docenti.unisr.it (L.P.); deodato.giulia7@gmail.com (G.D.); galatichiaramaria@gmail.com (C.M.G.); tecco.simona@hsr.it (S.T.); 2 Department of Clinical and Experimental Medicine, Titu Maiorescu University, 040051 Bucharest, Romania; doina.ghergic@prof.utm.ro

**Keywords:** tech neck, forward head posture, cranio-cervical posture, surface electromyography, portable electronic devices

## Abstract

**Highlights:**

**What are the main findings?**

- The HT+ operational group was associated with a relative predominance of anterior temporalis over masseter muscle recruitment in asymptomatic adults.
- The Activity Index decreased from the neutral head posture to anterior head flexion in both groups, whereas POC TA, POC MM, Asymmetry Index, Torque Index, and Impact remained comparatively stable after FDR correction.

**What are the implications of the main findings?**

- Anterior head flexion was associated with a shift in the relative contribution of the anterior temporalis and masseter muscles, rather than with broader changes across the evaluated masticatory sEMG indices.
- Cranio-cervical posture should be considered as a relevant contextual factor when interpreting masticatory sEMG patterns in asymptomatic individuals.

**Abstract:**

Background: The widespread use of portable electronic devices may involve prolonged exposure to altered cranio-cervical postures. This study investigated whether participants classified as HT+ and HT− according to a predefined operational classification differed in masticatory muscles recruitment. Methods: This cross-sectional study included 50 healthy adults (30 F, 20 M; mean age, 27 ± 5 years) classified into two operational groups. The HT+ group (*n* = 25) was defined by the simultaneous presence of portable electronic device use >8 h/day and habitual marked anterior head flexion, whereas the HT− operational group (*n* = 25) included participants who did not simultaneously meet both criteria. Participants underwent surface electromyography of the masseter and anterior temporalis muscles during maximum voluntary clenching (MVC) in the neutral head posture and during anterior head flexion (approximately target range 40–60°) using the MyoWise device (MyoWise, Via Gran Sasso 18, 20008 Bareggio, Milan, Italy). Percentage Overlapping Coefficients, Asymmetry Index, Activity Index, Torque Index, and Impact were assessed. Between-group comparisons, within-group posture-related changes, and between-group comparisons of change scores (Δ) were performed using parametric or non-parametric tests according to data distribution. The between-group comparison of the Activity Index in the reference posture was predefined as the primary analysis, whereas secondary analyses were adjusted for multiple comparisons using the Benjamini–Hochberg false discovery rate (FDR) procedure. Statistical significance was set at *p* < 0.05 for the primary analysis and at FDR-adjusted *q* < 0.05 for secondary analyses. Results: The Activity Index was significantly more negative in HT+ than in HT− participants in both the reference posture (*p* < 0.001) and during anterior head flexion (*p* = 0.003; *q* = 0.032). The Activity Index decreased significantly from the neutral head posture to anterior head flexion in both groups (HT+: *q* = 0.025; HT−: *q* = 0.002), whereas the magnitude of this change did not differ significantly between groups (*p* = 0.101; *q* = 0.607). No other sEMG outcome remained significant after FDR correction. Conclusions: The HT+ operational group was associated with a distinct temporalis–masseter recruitment pattern in asymptomatic adults. The Activity Index emerged as the sEMG parameter most strongly associated with operational HT classification, indicating that the observed between-group differences primarily involved the relative recruitment balance between the jaw-elevator muscles.

## 1. Introduction

Portable electronic devices have become integral to education, professional activities, communication, and leisure, resulting in a substantial increase in the amount of time spent interacting with smartphones, tablets, and laptop computers [1]. The use of these devices frequently requires the visual target to be positioned below eye level, encouraging prolonged anterior and downward head flexion, often accompanied by lower cervical flexion, upper cervical extension, anterior translation of the head, and increased thoracic kyphosis [2]. This postural configuration is commonly described as “tech neck” or “text neck” to describe the combination of postural alterations and cervico-scapular symptoms associated with prolonged portable electronic device use [3].

Recent studies have documented device-related cervical postural and functional findings in adolescents and young adults. Fontenele et al. reported cervical postural alterations among adolescents during smartphone use, particularly in participants presenting higher levels of smartphone addiction [4]. Elvan et al. subsequently observed that university students using mobile phones for at least four hours per day reported greater neck pain and showed reduced cervical flexor endurance compared with individuals with shorter daily exposure [5]. From a biomechanical perspective, increasing head and neck flexion has been associated with greater cervical extensor muscle activity [6] and increased gravitational demand on the cervical musculature [7], whereas ergonomic conditions that reduce cranio-cervical flexion may decrease biomechanical loading [8]. Taken together, these observations indicate that the biomechanical exposure associated with portable electronic device use depends not only on exposure duration but also on the cranio-cervical posture maintained during use [6,7,8].

The relevance of cranio-cervical posture may extend beyond the cervical region because the cranio-cervical and cranio-mandibular systems are functionally interconnected. Forward head posture has been shown to modify the electromyographic activity of the supra- and infrahyoid muscles during mouth opening [9], whereas submaximal jaw clenching is accompanied by co-activation of jaw and cervical muscles [10]. These observations support a functional coupling between cervical and mandibular motor systems [9,10]. Consistent with this relationship, a recent investigation reported measurable changes in condylar and incisal position during anterior head flexion resembling the posture commonly adopted during portable electronic device use [11]. Together, these findings provide a biomechanical and neuromuscular rationale for investigating whether cranio-cervical posture is also associated with differences in masticatory muscle recruitment [9,10,11].

Previous electromyographic studies have investigated the relationship between cranio-cervical posture and masticatory muscle recruitment, although the available findings are heterogeneous [12,13,14,15]. Tecco et al. reported significant associations between cranio-cervical postural parameters and the sEMG activity of several masticatory and cervical muscles [12]. Ballenberger et al. found that different upper cervical positions significantly influenced masseter activity during maximal clenching, whereas no significant effect was observed for the anterior temporalis muscle [13]. Similarly, Gadotti et al. reported posture-related differences in masseter activity during chewing under an experimentally induced forward head posture, whereas anterior temporalis activity did not show a comparable change; moreover, no significant differences in either muscle were identified when participants were classified according to their habitual upright or forward head posture [14]. Xu et al. also reported that head and neck posture influenced masticatory muscle electromyographic amplitude under different experimental conditions [15]. Taken together, these findings indicate that the relationship between cranio-cervical posture and masticatory-muscle recruitment may depend on the muscle examined, the functional task, the study population, and the experimental postural condition [12,13,14,15].

Surface electromyography (sEMG) provides a non-invasive approach for assessing the functional activity of superficial masticatory muscles. Normalization of the electromyographic signal against standardized reference contractions improves the comparability and interpretation of masticatory sEMG recordings [16]. Standardized indices derived from normalized recordings can characterize complementary aspects of masticatory-muscle organization. The Percentage Overlapping Coefficient (POC) describes the bilateral distribution of activity between homologous muscles [17], whereas the Asymmetry Index (ASIM) characterizes right–left predominance [18]. The Activity Index (ACT) reflects the relative contribution of the anterior temporalis and masseter muscles [17,18], the Torque Index (TOR) describes the relative activation balance between contralateral anterior temporalis–masseter muscle pairs, and Impact represents total standardized muscular activity [17]. Reference data for masticatory muscle activity and standardized sEMG indices have been reported in healthy young adults [17,19]. Together, these indices allow complementary characterization of masticatory muscle recruitment beyond raw electromyographic amplitude alone.

Despite growing interest in the musculoskeletal consequences of portable electronic device use, limited evidence is available regarding whether different patterns of device use and habitual cranio-cervical posture are associated with differences in masticatory muscle recruitment in asymptomatic adults. Moreover, it remains unclear whether participants with different combinations of these characteristics exhibit different changes in masticatory sEMG indices when experimentally placed in anterior head flexion. Evaluating normalized sEMG indices under both the reference posture and anterior head flexion conditions may therefore help characterize differences associated with the operational HT classification and changes associated with the experimental postural condition.

Accordingly, the present study aimed to compare normalized sEMG indices of the bilateral anterior temporalis and masseter muscles between asymptomatic adults classified as HT+ and HT− according to a predefined operational classification based on daily device-use duration and self-reported habitual cranio-cervical posture during device use. Recordings were obtained in the reference posture and during anterior head flexion. The specific objectives were to evaluate: (1) differences in masticatory muscle activity between the HT+ and HT− groups under the investigated conditions; (2) within-subject changes in the electromyographic indices between the neutral head posture and anterior head flexion; and (3) whether the magnitude of these within-subject changes between experimental conditions differed between groups. The HT+ group was defined by the simultaneous presence of prolonged portable electronic device use (>8 h/day) and self-reported habitual marked anterior head flexion, whereas the HT− group included participants who did not simultaneously meet both criteria. This classification was developed specifically for the present exploratory study and should be interpreted solely as an operational grouping variable rather than as a validated measure of habitual cranio-cervical posture, a clinical classification, or a predictor of cervical disease or temporomandibular disorders.

The null hypothesis was that no significant differences would be observed between participants classified as HT+ and HT− according to the predefined operational classification in any normalized sEMG index recorded in the reference posture; that anterior head flexion would not significantly modify the electromyographic indices within either group; and that the magnitude of the posture-related electromyographic changes would not differ between the two groups.

## 2. Subjects and Methods

### 2.1. Study Design and Ethical Approval

This comparative cross-sectional study was approved by the Ethics Committee of IRCCS San Raffaele Hospital, Milan, Italy (approval no. “parere09/int/2023,” dated 25 January 2023). The study was conducted in accordance with the principles of the Declaration of Helsinki and its subsequent amendments, as well as the International Council for Harmonisation guidelines for Good Clinical Practice (ICH-GCP). The manuscript was prepared in accordance with the Strengthening the Reporting of Observational Studies in Epidemiology (STROBE) Statement [20]. The completed STROBE checklist is available as Supplementary File S1.

### 2.2. Participant Selection and Group Classification

The final study sample comprised a total of 50 adult volunteers (30 F, 20 M; mean age, 27 ± 5.4 years) recruited at the Department of Dentistry, Vita-Salute San Raffaele University, Milan, Italy. The recruitment followed a sequential screening procedure in which predefined eligibility criteria were progressively assessed until the prespecified sample of 50 participants without temporomandibular disorders (TMDs) was reached. Individuals who did not meet the eligibility requirements did not proceed to the subsequent study procedures.

Participants were considered eligible if they met all the following inclusion criteria: age ≥ 18 years; Angle Class I molar and canine relationships; ability to understand and follow the operators’ instructions; and complete permanent dentition without missing teeth, except for the maxillary and mandibular third molars. The restriction to participants with Angle Class I dental relationships was intended to reduce occlusal and craniofacial heterogeneity and thereby limit a potential source of confounding in the assessment of posture-related differences in masticatory muscle activity.

Participants were excluded if any of the following criteria were present: ongoing orthodontic treatment with fixed appliances or clear aligners; coexisting clinically diagnosed musculoskeletal disorders potentially affecting posture or neuromuscular control, including clinically relevant cervical spine conditions; clinically evident global postural alterations or marked postural asymmetries identified during the preliminary clinical screening; a clinical diagnosis of temporomandibular disorder according to the Diagnostic Criteria for Temporomandibular Disorders (DC/TMD) [21]; Angle Class II or Class III dental malocclusion; transverse dental malocclusion; ongoing treatment with muscle relaxants, including botulinum toxin injections into the masseter muscles; use of analgesics or other medications potentially affecting neuromuscular activity; occlusal instability.

As part of the clinical eligibility screening, all participants underwent a qualitative global postural assessment in their habitual relaxed standing position using a postural analysis device (posturometer) consisting of a vertical plumb line, a reference grid, and a graduated rotating platform. The purpose of this assessment was to identify clinically evident global postural asymmetries or musculoskeletal alterations that could potentially interfere with posture, motor control, or surface electromyographic recordings. Participants in whom the screening raised suspicion of a relevant global postural alteration or marked asymmetry were not included in the study.

The posturometer was therefore used exclusively as a clinical screening and eligibility tool and was not intended to diagnose specific cervical conditions or to classify participants into the HT+ and HT− groups. Cranio-cervical alignment was assessed qualitatively, including observation relative to the Barré vertical line; however, no quantitative measurements of the craniovertebral angle, cervical lordosis, or other radiographic or instrumental cervical parameters were obtained. Consequently, minor variations observed during screening could not be interpreted as being outside physiological limits or as evidence of forward head posture, cervical straightening, or other specific cervical alterations. Participants presenting only minor findings that were not considered clinically relevant for exclusion remained eligible for the study.

Following confirmation of clinical eligibility, participants were allocated to two study groups, independently of the posturometer findings, according to a predefined operational classification combining self-reported daily portable electronic device use and habitual cranio-cervical posture during device use. Each participant completed an illustrated questionnaire developed by the research team specifically for the present study. The questionnaire was used as a structured operational tool to collect information on habitual posture, daily device-use duration, physical activity, and neck pain; it was not intended as a diagnostic or psychometric instrument, and no total or composite score was calculated. The complete questionnaire is provided as Supplementary File S2. The questionnaire has not undergone formal validation against objective instrumental measures of habitual cranio-cervical posture, and its test–retest reliability or reproducibility has not been formally assessed.

Habitual cranio-cervical posture was assessed through Questions 7, 8, and 9, which required participants to select, from five illustrated configurations, the posture that best represented the one maintained for the longest time during device use while standing, while seated, and during computer use, respectively. Daily portable electronic device use was assessed through Question 13 using the predefined categories 0–4 h/day, 5–8 h/day, and >8 h/day.

Physical activity (Questions 10 and 11) and self-reported neck pain during electronic device use (Question 14) were collected exclusively for descriptive purposes and were not used for group allocation or considered study outcomes. Neck pain was recorded only as a dichotomous anamnestic variable (presence/absence); its intensity, duration, frequency, associated disability, and specific clinical diagnosis were not assessed using validated clinical instruments.

Participants were classified as HT+ only when both of the following criteria were simultaneously met: (1) daily portable electronic device use >8 h/day; and (2) selection of a marked anterior head flexion posture, corresponding to postures 3, 4, or 5, in all three postural questions (Questions 7, 8, and 9). Participants were classified as HT− when they did not simultaneously meet both HT+ criteria. Thus, participants reporting 5–8 h/day did not meet the >8 h/day device-use criterion, whereas participants reporting >8 h/day were classified as HT− if at least one of the three postural questions indicated a relatively neutral cranio-cervical posture corresponding to posture 1 or 2. Device-use duration alone was therefore not sufficient for HT+ classification. Because HT− was defined by the absence of simultaneous fulfillment of both HT+ criteria, this group could include different combinations of device-use duration and habitual posture and was therefore potentially heterogeneous with respect to the two classification components.

The >8 h/day threshold was defined a priori as an operational criterion for particularly prolonged daily exposure and should not be interpreted as a validated biological or clinical cut-off. Similarly, the distinction between illustrated postures 1–2 and 3–5 was defined a priori as an operational categorization for the present study and has not been externally validated as a threshold distinguishing different levels of biomechanical exposure.

Because a systematic log of individuals who failed the eligibility criteria before inclusion in the study sample was not maintained, the total number initially screened and stage-specific exclusion counts could not be reliably reconstructed retrospectively.

**Figure 1:**
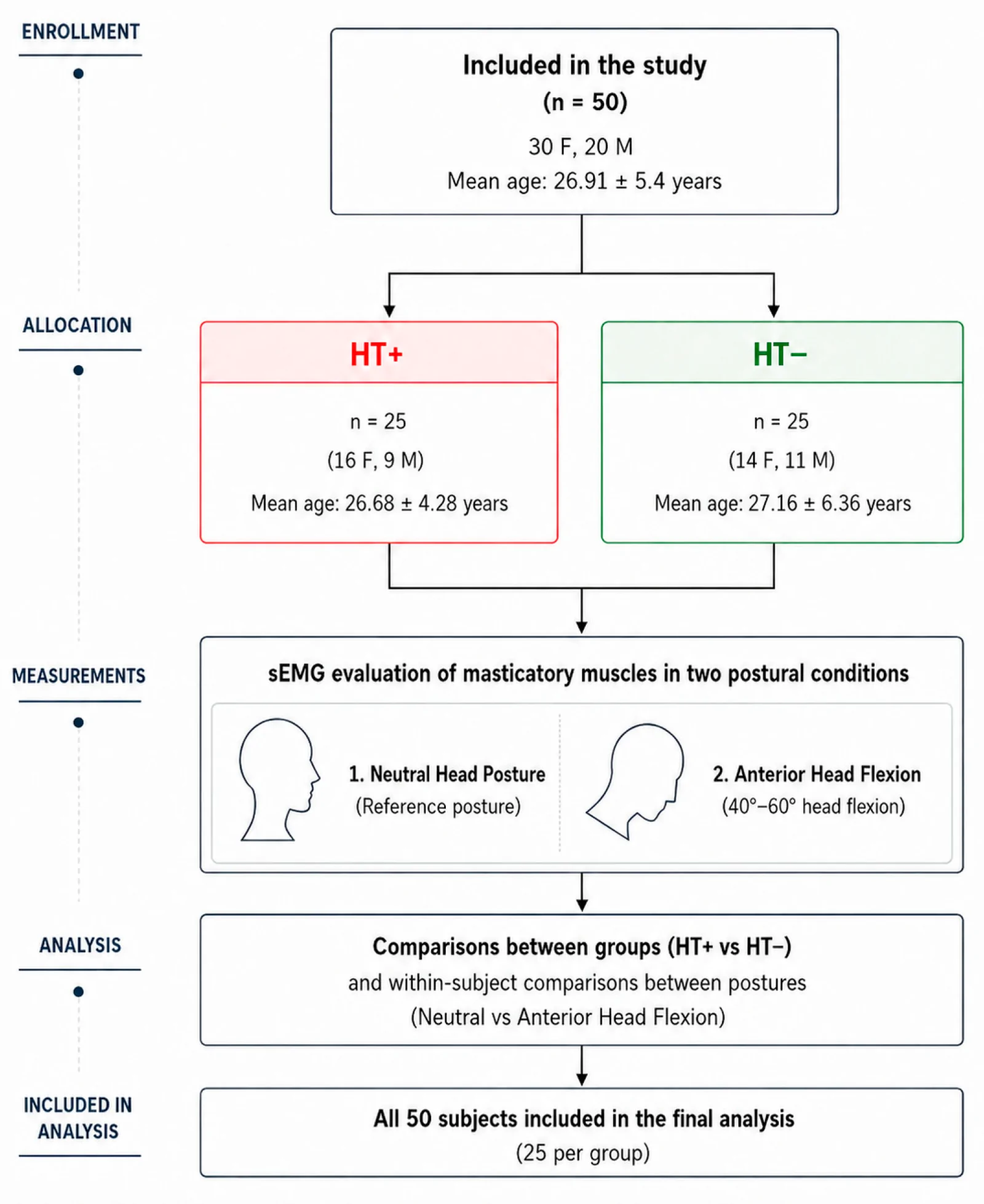
Study flowchart reporting participant allocation.

The HT+ group consisted of 25 participants, including 16 females and 9 males, with a mean age of 26.68 ± 4.28 years. Similarly, the HT− group comprised 25 participants, including 14 females and 11 males, with a mean age of 27.16 ± 6.36 years. The distribution of participants by study group, sex, and age is summarized in Figure 1.

### 2.3. Sample Size Justification

An a priori pilot-based sample size calculation was performed using G*Power software (version 3.1; Heinrich Heine University, Düsseldorf, Germany) for the primary electromyographic outcome, Activity Index, considering the planned between-group comparison in the reference posture. The calculation was based on preliminary data from the first 10 enrolled participants, equally distributed between the HT+ and HT− groups, which showed an approximate between-group difference of 19.5% in the Activity Index in the reference posture. Using these preliminary data, with a two-sided alpha level of 0.05, a statistical power of 0.80, and equal group allocation, the minimum required sample size was estimated at 7 participants per group. Because estimates derived from small pilot samples may be unstable, the final target sample size was conservatively increased to 25 participants per group to improve the reliability, precision, and robustness of the between-group comparisons. Therefore, the final cohort of 50 participants exceeded the minimum sample size indicated by the pilot-based power analysis.

### 2.4. Surface Electromyographic Assessment Protocol

All patients underwent surface electromyographic assessment using a MyoWise system (MyoWise, Via Gran Sasso 18, 20008 Bareggio, Milan, Italy), which was used for signal acquisition and analysis. The MyoWise sensors acquired the sEMG signals at a sampling frequency of 1000 Hz, with an acquisition bandwidth of 10–500 Hz. The system incorporates first-order hardware high-pass and low-pass filters with a slope of −20 dB/decade.

To improve the interpretability of the surface electromyographic signal and minimize the influence of potential postural confounders, a standardized postural protocol was adopted during both the calibration and sEMG recording phases [22]. Patients were seated on a rigid, non-reclining chair to prevent alterations in axial loading. The pelvis and trunk were maintained in a stable position, with the knees flexed at approximately 90° and both feet fully supported on the floor. The trunk was maintained in a physiological upright posture without voluntary compensatory adjustments. Neutral cranio-cervical alignment was established with the Frankfurt horizontal plane parallel to the floor. The same postural conditions were rigorously reproduced during each calibration and recording session.

Before electrode application, eyeglasses and any accessories that could interfere with electrode positioning were removed. The skin was inspected to exclude local conditions that could compromise electrode adhesion, including skin lesions or adhesive devices applied over the recording areas. General procedures for surface electromyographic acquisition were based on the SENIAM guidelines for Surface Electromyography for the Non-Invasive Assessment of Muscles [23], particularly with regard to skin preparation, bipolar surface-electrode configuration, electrode orientation relative to the predominant muscle-fibre direction, electrode fixation, and standardization of recording conditions.

To reduce skin–electrode impedance, the skin surface was carefully cleaned with 90% denatured ethyl alcohol before electrode application, thereby improving electrode adhesion and electrical signal conduction. Disposable pre-gelled bipolar surface electrodes (Kendall, Cardinal Health™, Dublin, OH, USA), measuring 30 × 24 mm, were applied to the skin overlying the muscles of interest.

Muscle-specific electrode placement over the masseter and anterior temporalis muscles was performed according to previously described standardized procedures for masticatory sEMG [24,25]. For electrode placement over the masseter muscle (MM), the operator identified the superficial muscle belly by palpation during dental clenching. The electrode was positioned over the palpated muscle belly and aligned parallel to the predominant direction of the muscle fibres. Electrode positioning was based on the anatomical region defined by the exocanthion–gonion and tragus–labial commissure reference lines, consistent with standardized masticatory sEMG methodology [24,25]. For the anterior temporalis muscle (TA), the muscle belly was identified by palpation during dental clenching. The electrode was positioned vertically along the anterior margin of the muscle, approximately corresponding to the frontoparietal suture, according to previously described standardized masticatory sEMG procedures [24].

Palpation of the muscle belly during clenching and alignment of the bipolar electrodes with the predominant muscle-fibre direction were used to improve the anatomical consistency and selectivity of electrode placement and to reduce potential cross-talk from neighbouring muscles. Nevertheless, as is inherent to surface electromyography, complete elimination of cross-talk cannot be guaranteed.

After electrode application, gentle bilateral pressure was applied to facilitate adhesion of the conductive gel to the skin surface and optimize the quality of the skin–electrode contact [26]. The examination was conducted in the presence of two operators to minimize human error and ensure independent verification throughout the different phases of the procedure.

All sEMG recordings were performed by the same trained and calibrated operator to minimize inter-operator variability. The operator responsible for sEMG acquisition was unaware of the participants’ HT+/HT− group allocation at the time of recording. Information used for HT+/HT− classification was managed separately and was not disclosed to the sEMG operator. Furthermore, sEMG assessments were performed during routine clinical hours, approximately between 10:00 and 18:00. Both postural conditions were recorded within the same experimental session for each participant, thereby ensuring identical temporal conditions for the within-subject comparison. Device configuration and calibration were carried out in strict accordance with the manufacturer’s instructions.

Before recording, each patient received a detailed explanation of the electromyographic procedure. Patients were instructed not to adjust or displace the cotton rolls once they had been inserted. They were asked to look straight ahead, maintain an upright trunk position without leaning against the backrest, and avoid changing position unless specifically instructed by the operator. Upon the operator’s command, patients were required to clench on the cotton rolls and maintain a constant contraction force throughout the 5 s recording period. Verbal cues were provided continuously by the operator to help patients maintain attention and avoid variations in contraction intensity. Patients were also instructed to avoid contracting the facial expression muscles during clenching and, once the recording had been completed, not to displace or drop the cotton rolls but to wait until they were removed by the operator.

For the reference calibration, two standardized cotton rolls, each measuring 10 mm in diameter, were positioned bilaterally between the mandibular second premolar and first molar. Participants performed a 5 s maximum voluntary clench under this standardized condition. The sEMG activity recorded during cotton-roll clenching was used as the reference for normalization of the subsequent experimental recordings, according to established standardized masticatory sEMG methodology [16,17,25].

**Figure 2:**
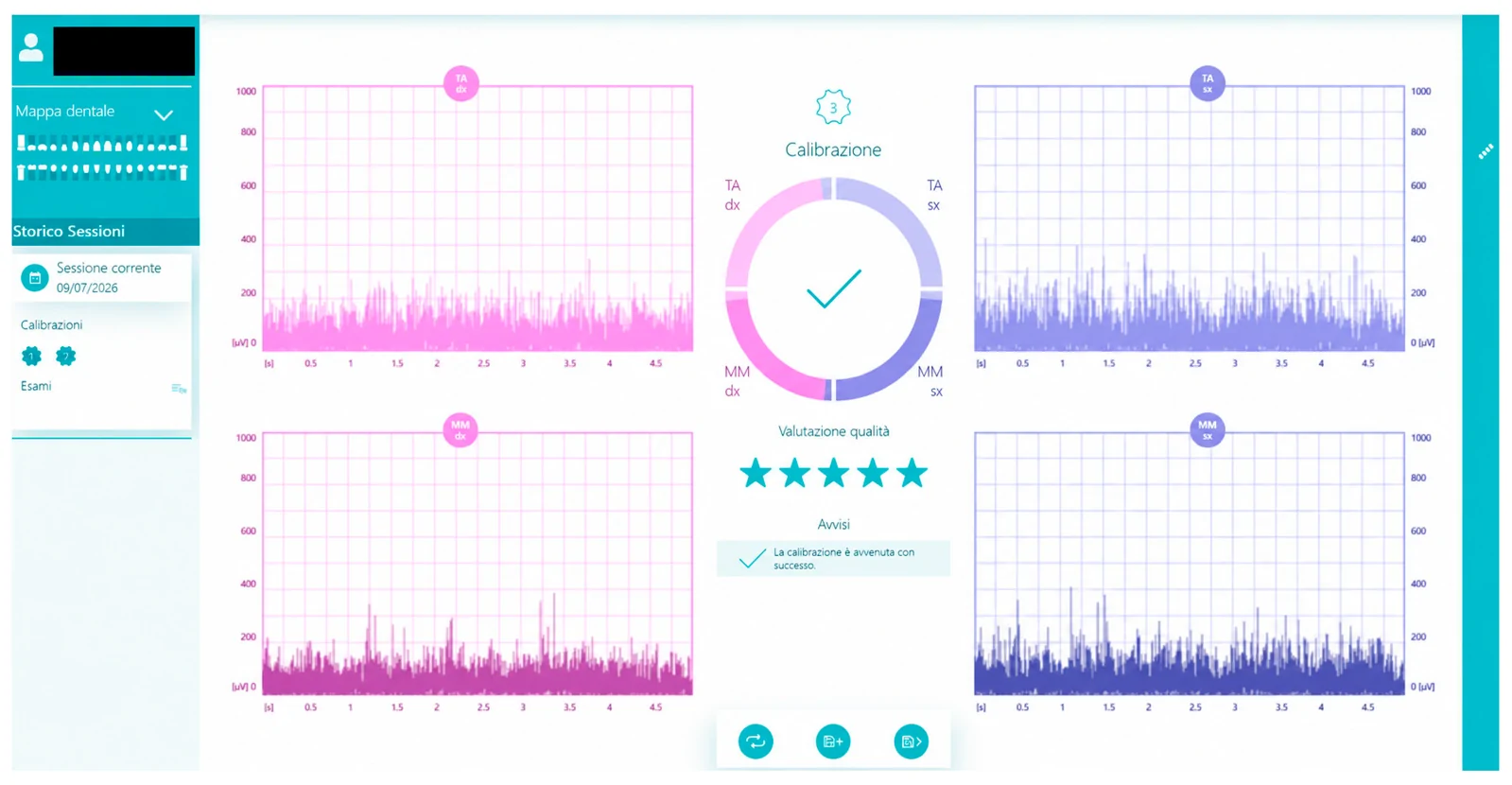
Surface electromyographic (sEMG) calibration of the bilateral anterior temporalis (TA) and masseter (MM) muscles. The interface displays the acquired muscle signals and the software-based assessment of calibration quality. Light pink and dark pink/purple elements correspond to the right TA and MM muscles, respectively, whereas light blue/lavender and dark blue elements correspond to the left TA and MM muscles, respectively. The software interface is displayed in Italian; the relevant labels are translated as follows: “Mappa dentale”, dental map; “Storico Sessioni”, session history; “Sessione corrente”, current session; “Calibrazioni/Calibrazione”, calibrations/calibration; “Esami”, examinations; “Valutazione qualità”, quality assessment; “Avvisi”, alerts; and “La calibrazione è avvenuta con successo”, calibration was completed successfully. The abbreviations “dx” and “sx” indicate right and left, respectively. Patient-identifying information was masked to preserve confidentiality.

The MyoWise software (version 1.4; MyoWise, Via Gran Sasso 18, 20008 Bareggio, Milan, Italy) allows multiple calibration recordings to be performed and subsequently selected for analysis. The quality of each calibration was automatically assessed by the software using a rating scale ranging from one to five stars. Only calibrations receiving a score of at least four stars were considered acceptable and retained for analysis. The software also identified potential abnormalities in the execution of the clenching task, including variations in contraction intensity. When such abnormalities were detected, the calibration recording was discarded and repeated. At least two acceptable calibration recordings were retained for each patient (Figure 2).

Following calibration, the cotton rolls were removed and the experimental sEMG recordings were performed on the natural dentition during maximal voluntary clenching (MVC) in maximum intercuspation. Thus, the experimental signals analysed in the present study were not recordings of resting or basal muscle activity. For each experimental acquisition, participants were instructed to reach and maintain a continuous MVC throughout a standardized 5 s recording period.

Experimental sEMG recordings were obtained under two postural conditions: neutral head posture (reference posture) and anterior head flexion, with one 5 s recording acquired and retained for each condition. The order of the two experimental conditions was fixed for all participants, with the neutral head posture always assessed first and anterior head flexion second; the order was not randomized or counterbalanced. The first 5 s MVC recording was performed in the neutral head posture. After completion of this recording, a 120 s recovery interval was allowed to minimize the potential influence of muscle fatigue. This interval was selected because recovery time may influence neuromuscular responses and electromyographic signal characteristics, and 120 s rest periods have previously been used to assess recovery following isometric contractions of the mandibular elevator muscles [27,28]. Participants then assumed the anterior head flexion condition. Once the predefined target position had been reached, a 10 s stabilization period was allowed before sEMG acquisition. During this interval, participants were instructed to maintain the prescribed body and cranio-cervical position without voluntary adjustments. At the end of the stabilization period, a second observer verified maintenance of the predefined experimental position. Once the position had been confirmed, participants repeated the same 5 s MVC task in maximum intercuspation, without removing or repositioning the electrodes.

During the second condition, participants were instructed to adopt marked anterior head flexion within a predefined approximate target range of 40–60°, intended to reproduce a postural condition commonly observed during portable electronic device use [29]. The use of an approximate angular range rather than a single fixed value was intended to provide a practical standardized target for the experimental condition while accommodating interindividual anatomical and anthropometric variability. However, because the individual flexion angle was not instrumentally quantified, equivalence of the actual cranio-cervical position across participants could not be verified. Accordingly, some interindividual variability in the actual cranio-cervical position during this condition cannot be excluded.

**Figure 3:**
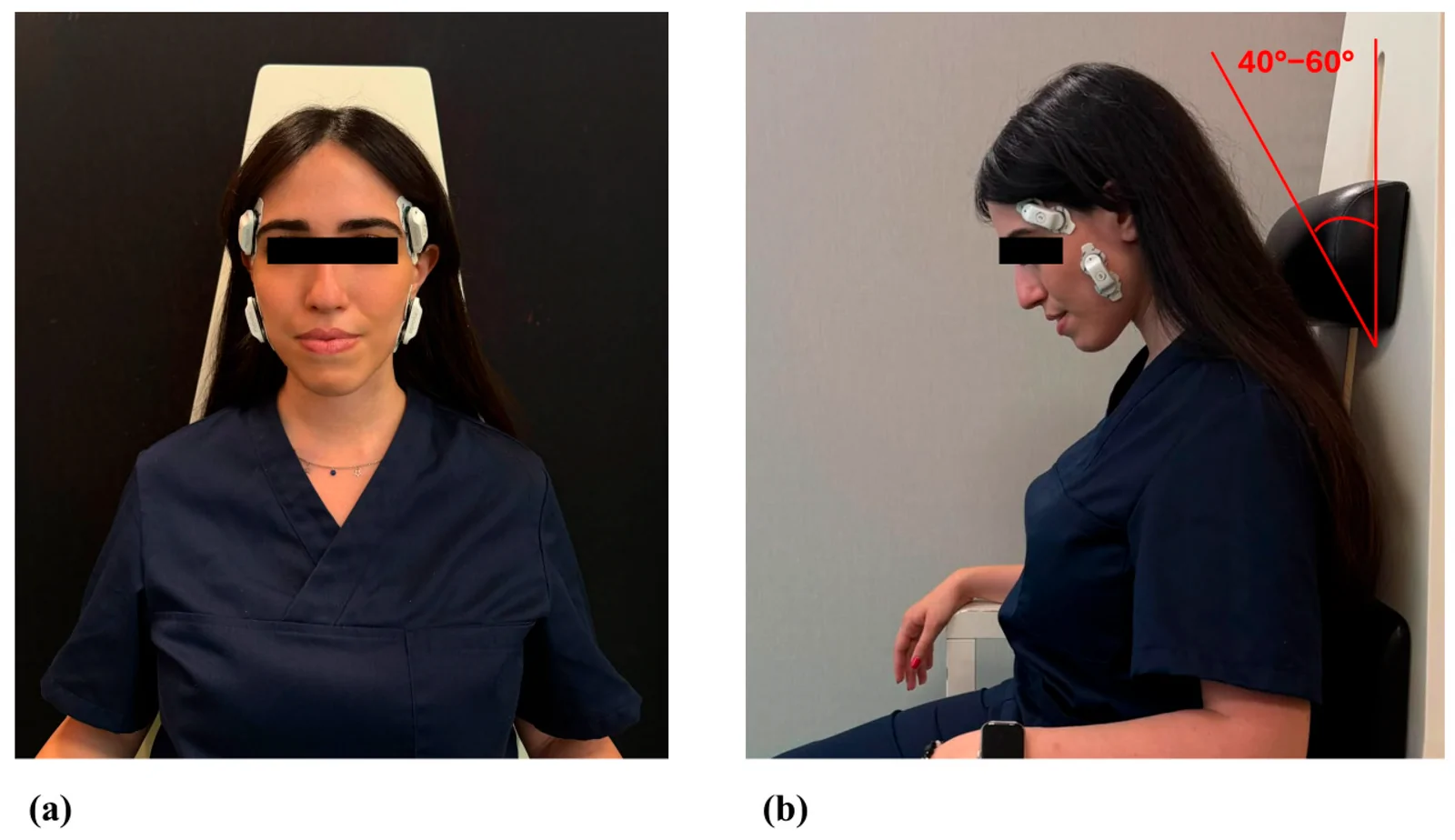
Surface electromyographic assessment performed under the two experimental postural conditions. (**a**) sEMG recording in the habitual neutral head posture; (**b**) sEMG recording during standardized anterior head flexion, corresponding to an approximate target range of 40–60°.

To improve procedural consistency, all recordings were performed using the same rigid chair, with the seated position and trunk alignment maintained unchanged. Anatomical and postural reference points, including the tragus and acromion, were used to guide positioning. A second observer, distinct from the operator responsible for sEMG acquisition, verified maintenance of the predefined body and cranio-cervical position throughout the recording. During anterior head flexion, the observer specifically verified that the participant remained within the approximate 40–60° experimental postural range. No inclinometer, digital goniometer, or other instrumental system was used to quantitatively measure the individual cranio-cervical flexion angle. Therefore, the 40–60° interval should be interpreted as an approximate experimental target range rather than as an instrumentally measured angle for each participant (Figure 3).

Thus, the muscular task, mandibular position, requested contraction intensity (MVC), recording duration, electrode configuration, verbal instructions, and acquisition procedure were maintained constant across the two experimental conditions, whereas cranio-cervical position represented the experimental condition intentionally modified.

No additional manual post-processing of the sEMG signals was performed by the investigators. Signal normalization and computation of the standardized indices were performed automatically through the proprietary MyoWise (MyoWise, Via Gran Sasso 18, 20008 Bareggio, Milan, Italy) processing pipeline. Specific internal parameters of the proprietary processing algorithm that are not disclosed in the manufacturer documentation, including the exact rectification, smoothing, and amplitude-estimation procedures, were therefore neither selected nor modified by the investigators.

The following standardized surface electromyographic indices were calculated and considered as outcome variables to characterize complementary aspects of masticatory muscle activity and recruitment:

- Percentage Overlapping Coefficient of the anterior temporalis muscles (POC TA, %): quantifies the degree of overlap between the normalized sEMG activity of the right and left anterior temporalis muscles recorded simultaneously during the same standardized task. In this context, “overlap” refers to the degree of correspondence between the two normalized activation profiles over the recording interval: the more similar the relative activation of the homologous muscles, the greater the overlap and the closer the POC value is to 100%. Conversely, increasing differences between the right- and left-side normalized activity reduce the overlap and therefore the POC value. POC TA thus reflects bilateral temporalis symmetry rather than absolute muscle force. Values range from 0% to 100%, with 100% representing complete overlap. Values above approximately 80% have been reported in healthy or asymptomatic individuals under standardized clenching conditions [17,25].
- Percentage Overlapping Coefficient of the masseter muscles (POC MM, %): quantifies the degree of overlap between the normalized sEMG activity of the right and left masseter muscles recorded simultaneously during the same standardized task. A greater correspondence between the two normalized activation profiles results in a higher percentage of overlap, indicating greater bilateral symmetry of masseter recruitment, whereas lower values reflect increasing differences in relative activation between the two sides. POC MM should therefore be interpreted as an index of bilateral neuromuscular symmetry rather than as a direct measure of muscle force or as a stand-alone diagnostic parameter. Values range from 0% to 100%, with 100% representing complete overlap. Values above approximately 80% have been reported in healthy or asymptomatic individuals under standardized clenching conditions [17,25]
- Asymmetry Index (ASIM, %): quantifies the relative predominance of the overall normalized activity of the right- versus left-side masticatory muscles. Values range from −100% to +100%; positive values indicate right-side predominance, negative values indicate left-side predominance, and values approaching 0% indicate greater bilateral balance. Values within approximately −10% to +10% have been reported as reference values in healthy or asymptomatic populations assessed under standardized conditions [18]
- Activity Index (ACT, %): describes the relative contribution of the anterior temporalis and masseter muscles to the overall standardized masticatory muscle activity. Values range from −100% to +100%; negative values indicate relative predominance of anterior temporalis activity, positive values indicate relative predominance of masseter activity, and values approaching 0% indicate a more balanced contribution of the two muscle groups. Values within approximately −10% to +10% have been reported in healthy or asymptomatic individuals under standardized clenching conditions [17,18]
- Torque Index (TOR, %): is an sEMG-derived index describing the relative activation balance between contralateral anterior temporalis–masseter muscle pairs. Values range from −100% to +100%; positive and negative values indicate opposite directions of cross-muscle predominance, whereas values approaching 0% indicate a more balanced contribution of the contralateral muscle pairs. The term “Torque Index” represents the established nomenclature of this parameter and, in the present study, should not be interpreted as a direct measure of mechanical torque, force, mandibular moment, displacement, or kinematics. Values within approximately −10% to +10% have been reported as reference values under standardized conditions [17,25]
- Impact (IMP, %): expresses the overall standardized masticatory muscle activity recorded during maximum voluntary clenching in maximum intercuspation relative to that obtained during the cotton-roll reference calibration. A value of 100% indicates an overall activity comparable with that recorded during the reference contraction, whereas values below or above 100% indicate relatively lower or higher standardized muscular activity, respectively. Values of approximately 80–120% have been reported as reference values under standardized clenching conditions [17,24]

These reference values were derived from healthy or asymptomatic populations examined under standardized normalization and clenching conditions and were used in the present study only to provide descriptive context. They were not applied as diagnostic cut-offs or to classify individual participants as having physiological or pathological muscle function.

**Figure 4:**
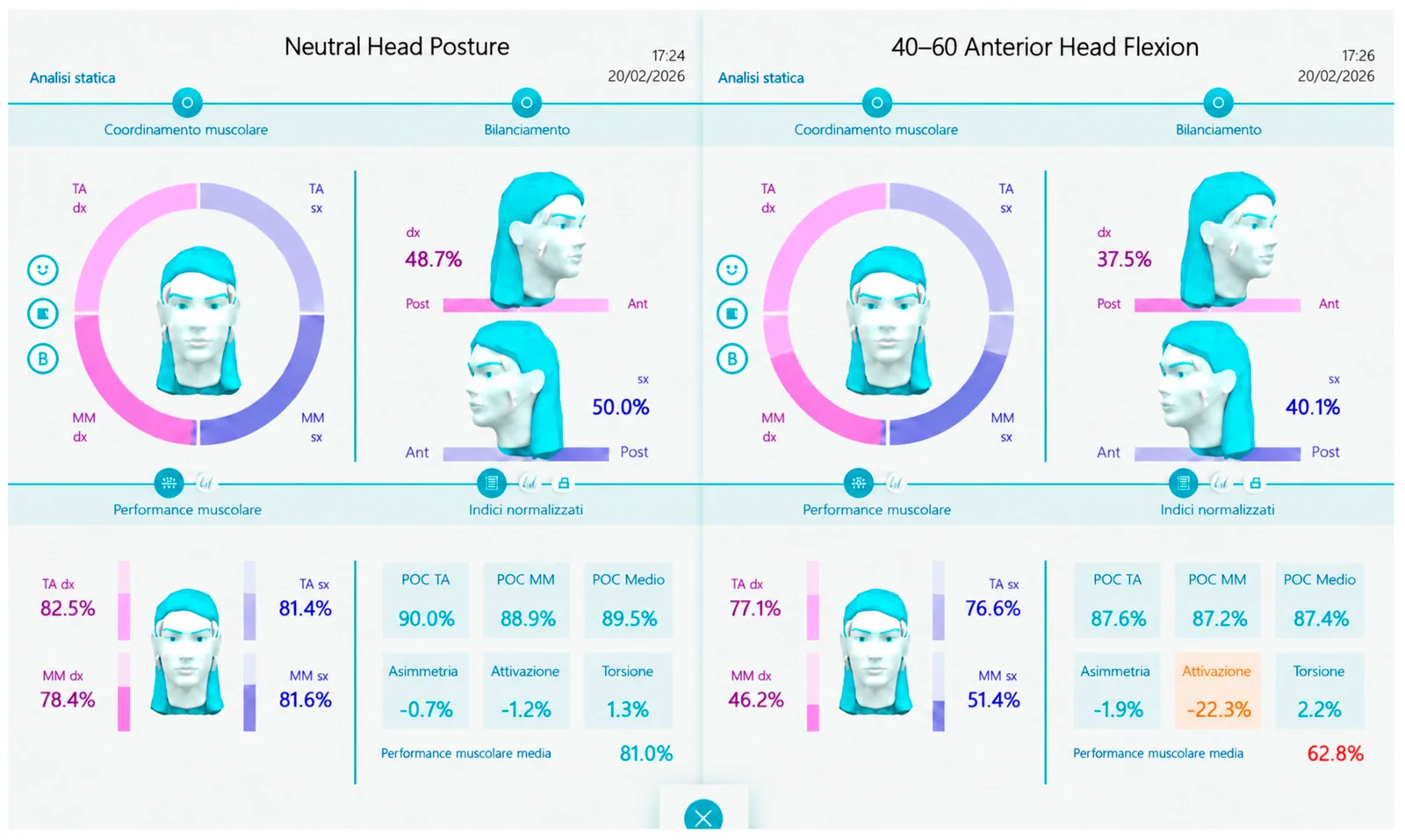
Representative software interface displaying the standardized surface electromyographic indices obtained during maximum voluntary clenching in maximum intercuspation under the two experimental postural conditions: neutral head posture (**left**) and anterior head flexion within the approximate 40–60° target range (**right**). The interface reports bilateral anterior temporalis (TA) and masseter (MM) muscle activity together with the derived Percentage Overlapping Coefficients (POC TA and POC MM), Asymmetry Index (ASIM), Activity Index (ACT), Torque Index (TOR), and Impact (IMP). Light pink and dark pink/purple elements correspond to the right TA and MM muscles, respectively, whereas light blue/lavender and dark blue elements correspond to the left TA and MM muscles, respectively; “dx” and “sx” denote the right and left sides, respectively. In this example, anterior head flexion is accompanied by a marked decrease in the Activity Index, from −1.2% to −22.3%, indicating a shift in the relative recruitment pattern toward greater anterior temporalis predominance over the masseter muscles.

A representative example of the software interface displaying the standardized sEMG indices obtained under the two experimental postural conditions is shown in Figure 4.

No formal intra-operator reliability analysis was performed for sEMG acquisition. Inter-observer reliability was not applicable to sEMG acquisition because all recordings were performed by a single operator; however, no formal reliability assessment was conducted for the second observer’s verification of the experimental postural condition.

### 2.5. Data Handling and Statistical Analysis

Data were processed using Microsoft Excel for descriptive statistical analysis, while inferential statistics was performed with StatPlus:mac Pro software (version 8; AnalystSoft Inc., Walnut, CA, USA).

Before statistical analysis, the dataset was inspected for data-entry errors and extreme values. Potential outliers were evaluated in relation to the original sEMG recordings and acquisition quality-control procedures. Observations were retained when no identifiable technical artefact or recording error was detected.

Normality was assessed using the Shapiro–Wilk test separately for each electromyographic variable and study group.

Surface electromyographic outcomes recorded in the neutral head posture and during anterior head flexion were first compared between the HT+ and HT− groups. Because the two groups consisted of independent participants, Welch’s independent-samples *t*-test was used when the outcome was normally distributed in both groups. When normality was not confirmed in at least one group, the Mann–Whitney U test was applied.

Within-subject changes between the neutral head posture and anterior head flexion were subsequently evaluated within each group. Since measurements in the neutral head posture and during 40–60° anterior head flexion were obtained from the same participants, paired analyses were performed. For each electromyographic outcome, the within-subject change was calculated as Δ = value during anterior head flexion − value in the reference posture. The normality of the paired differences was assessed using the Shapiro–Wilk test. Normally distributed differences were analysed using the paired-samples *t*-test, whereas non-normally distributed differences were assessed using the Wilcoxon signed-rank test.

To determine whether the magnitude of the within-subject change between the neutral head posture and anterior head flexion differed between the HT+ and HT− groups, change scores were compared between the two independent groups. Welch’s independent-samples *t*-test was used when the change scores were normally distributed in both groups, whereas the Mann–Whitney U test was applied when normality was not satisfied in at least one group. The predefined primary analysis was evaluated at a two-sided significance level of *p* < 0.05 without multiplicity adjustment. For secondary and exploratory analyses, multiplicity was addressed using the Benjamini–Hochberg false discovery rate procedure within predefined families of related comparisons, and statistical significance was determined on the basis of FDR-adjusted *q* < 0.05. Unadjusted *p*-values are reported alongside *q*-values for transparency, whereas interpretation of secondary analyses was based on the FDR-adjusted results. To complement the interpretation of the statistical significance of the observed differences, effect sizes were also reported. Cliff’s delta (δ) was used for comparisons between the independent HT+ and HT− groups, whereas the matched-pairs rank-biserial correlation (r_rb) was calculated for within-subject comparisons between the reference posture and the 40–60° anterior head flexion. Effect sizes were interpreted according to their absolute magnitude. For Cliff’s δ, values < 0.147 were considered negligible, 0.147–<0.33 small, 0.33–<0.474 moderate, and ≥0.474 large. For r_rb, absolute values < 0.10 were considered negligible, 0.10–<0.30 small, 0.30–<0.50 moderate, and ≥0.50 large. The sign of the effect-size estimate indicated the direction of the observed difference or change.

The primary analysis was defined a priori as the between-group comparison of the Activity Index in the neutral head posture. This outcome was selected as the primary electromyographic endpoint and was used for the a priori sample size calculation. Therefore, no multiplicity adjustment was applied to this predefined primary analysis.

Because additional electromyographic indices, postural conditions, within-group changes, and change-score comparisons were also evaluated, these analyses were considered secondary or exploratory. For these secondary analyses, multiplicity was addressed using the Benjamini–Hochberg false discovery rate procedure within predefined families of related comparisons. Effect sizes were reported to support the interpretation of the magnitude of the observed differences.

As a sensitivity analysis limited to the primary electromyographic outcome, Activity Index, a repeated-measures factorial model was additionally performed to directly assess the Group × Posture interaction. Group was included as the between-subject factor (HT+ vs. HT−), Posture as the within-subject factor (reference posture vs. anterior head flexion), and the Group × Posture term was used to evaluate whether the posture-induced change in Activity Index differed between groups.

No missing data were present, and all enrolled participants were included in the statistical analyses.

## 3. Results

Between-group comparisons of surface electromyographic outcomes between HT+ and HT− participants in the neutral head posture and during anterior head flexion are presented in Table 1.

No significant between-group differences were observed for POC TA, POC MM, Asymmetry Index, Torque Index, or Impact under either postural condition after correction for multiple comparisons. In contrast, the Activity Index showed a marked between-group difference. In the neutral head posture, the HT+ group exhibited substantially more negative Activity Index values than the HT− group (−17.9 [−30.3; −7.8] vs. −0.8 [−5.8; 3.3], respectively). The Hodges–Lehmann estimate of the between-group difference was −17.4 [95% CI: −27.1; −9.3], with a large effect size (Cliff’s δ = −0.688; *p* < 0.001). This comparison represented the predefined primary analysis and was therefore not adjusted for multiplicity.

During anterior head flexion, the Activity Index remained significantly more negative in the HT+ group than in the HT− group (−18.3 [−34.2; −13.4] vs. −10.2 [−16.1; −1.7], respectively). The Hodges–Lehmann estimate was −12.4 [95% CI: −23.1; −4.0], with a large effect size (Cliff’s δ = −0.493; *p* = 0.003; q = 0.032). Thus, the Activity Index was the only electromyographic outcome showing a significant between-group difference under both postural conditions.

Within-group comparisons between the neutral head posture and anterior head flexion are reported in Table 2.

Within-group changes from the neutral head posture to anterior head flexion are reported in Table 2. After FDR correction, the Activity Index was the only outcome showing a significant within-subject change in either group. In HT+ participants, ACT decreased from the reference posture to anterior head flexion (median Δ = −4.8 [−13.0; −1.4]; 95% CI: −11.2 to −1.4; r_rb = −0.637; *p* = 0.004; q = 0.025). ACT also decreased significantly in HT− participants (median Δ = −11.1 [−17.9; −5.9]; 95% CI: −15.8 to −6.6; r_rb = −0.809; *p* < 0.001; *q* = 0.002). Impact did not remain statistically significant after FDR correction (*q* = 0.095 in both groups), and no significant changes were identified for POC TA, POC MM, Asymmetry Index, or Torque Index.

Between-group comparisons of the within-subject changes between the neutral head posture and anterior head flexion are summarized in Table 3.

No significant between-group differences in Δ values were observed for any of the evaluated electromyographic indices. Specifically, the between-group difference in ΔACT was not statistically significant (Hodges–Lehmann difference in Δ, HT+ − HT− = 5.4 [95% CI: −0.8; 11.0]; Cliff’s δ = 0.272; *p* = 0.101; *q* = 0.607). No significant between-group differences were identified for POC TA, POC MM, Asymmetry Index, Torque Index, or Impact (all *q* ≥ 0.823).

As a sensitivity analysis for the primary electromyographic outcome, the repeated-measures factorial model confirmed a significant main effect of Group (F(1,48) = 16.445, *p* < 0.001) and Posture (F(1,48) = 15.720, *p* < 0.001), whereas the Group × Posture interaction was not significant (F(1,48) = 2.712, *p* = 0.106).

**Figure 5:**
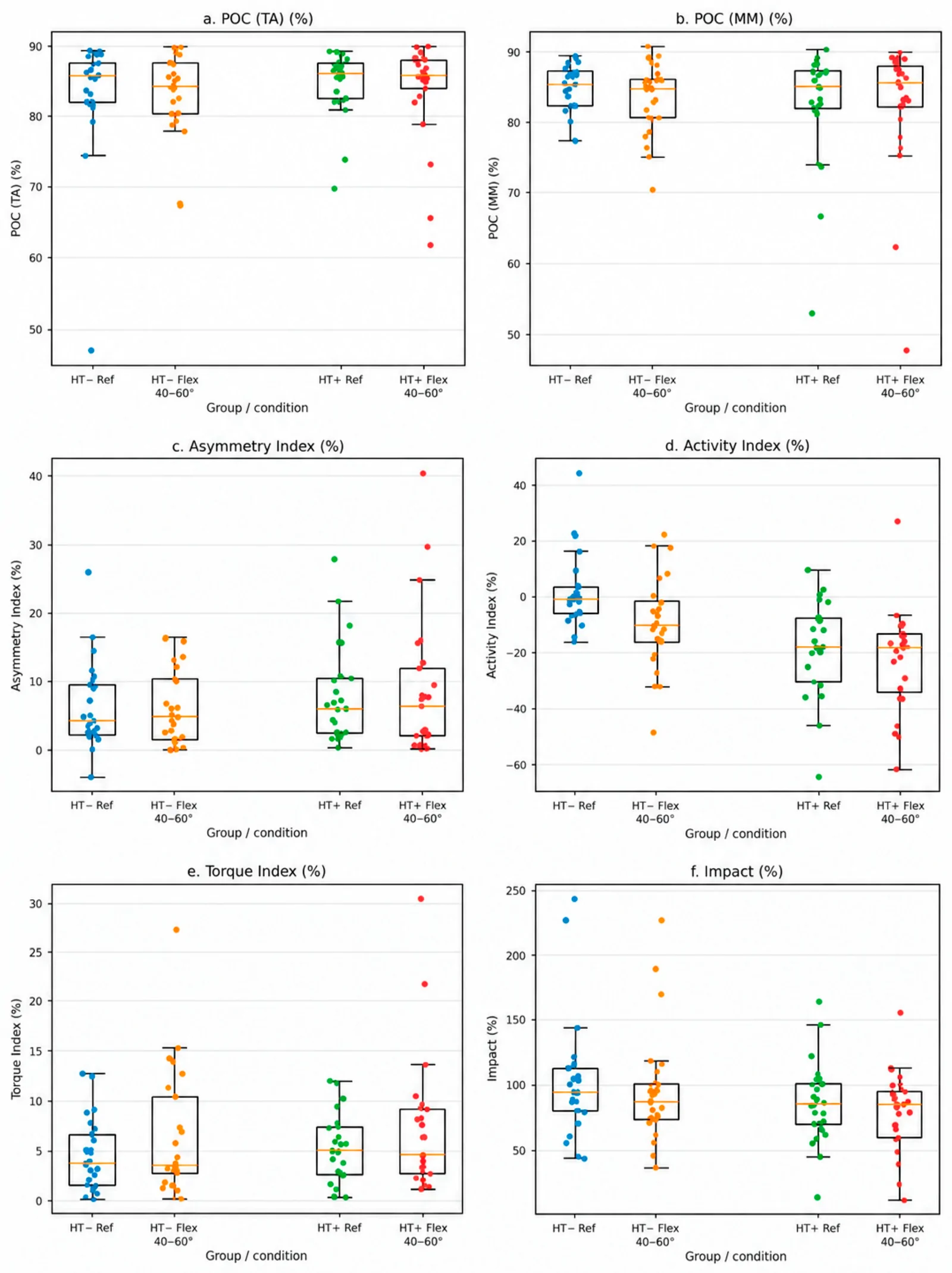
Distribution of surface electromyographic outcomes by operational HT classification and experimental posture. Boxplots with superimposed individual observations are shown for (**a**) percentage overlapping coefficient of the anterior temporalis muscles (POC TA), (**b**) percentage overlapping coefficient of the masseter muscles (POC MM), (**c**) Asymmetry Index (ASIM), (**d**) Activity Index (ACT), (**e**) Torque Index (TOR), and (**f**) Impact (IMP) in the HT− and HT+ groups under the reference posture and anterior head flexion (40–60°). Boxes represent the interquartile range, horizontal lines indicate the median, whiskers extend to the most extreme observations within 1.5 times the interquartile range, and individual points represent single participants.

Individual data distributions and between-subject variability for all electromyographic outcomes under both postural conditions are illustrated in Figure 5.

## 4. Discussion

The present study was designed to compare the neuromuscular activity of the masticatory muscles between clinically asymptomatic individuals classified as HT+ and HT− according to a predefined operational classification, both in the reference posture and during anterior head flexion. The main finding of the present study was that the operational HT classification was associated with a selective difference in relative temporalis–masseter recruitment, rather than with broader differences across the other evaluated sEMG indices. The Activity Index was markedly more negative in HT+ than in HT− participants in the neutral head posture, with a large between-group effect (Cliff’s δ = −0.688), and this difference persisted during anterior head flexion, again with a large effect (Cliff’s δ = −0.493). In contrast, POC TA, POC MM, Asymmetry Index, Torque Index, and Impact showed no robust between-group differences after correction for multiple comparisons. Taken together, these findings suggest that the association with operational HT classification primarily involved the relative contribution of the anterior temporalis and masseter muscles rather than broader differences across the other evaluated sEMG indices.

The available evidence on the relationship between cranio-cervical posture and masticatory function remains heterogeneous. Gadotti et al. reported posture-related changes in masticatory-muscle activity during chewing, with a significant increase in masseter activity during experimentally induced forward head posture, whereas the corresponding change in anterior temporalis activity did not reach statistical significance. In addition, no significant differences were observed when participants were compared according to their habitual upright or forward head posture [14]. Using a different physiological outcome, Yao et al. found no significant differences in masseter or temporalis pressure pain thresholds between patients with temporomandibular disorders with and without forward head posture, and no significant association between forward head posture and masticatory-muscle pressure pain thresholds was identified [30]. These findings highlight that the effects associated with cranio-cervical posture may depend on the specific physiological construct examined. Electromyographic activity, the relative muscle recruitment, and pressure pain sensitivity represent distinct dimensions of masticatory function and should not necessarily be expected to exhibit parallel changes. Differences in study populations, the presence or absence of temporomandibular disorders, the definition and assessment of head posture, the motor task performed, and the recording methodology may further contribute to the variability of findings across studies. Accordingly, the present results should be interpreted within this heterogeneous methodological and physiological context rather than as evidence of a uniform effect of cranio-cervical posture on masticatory function.

Within this heterogeneous framework, the Activity Index (ACT) was the parameter most consistently associated with the operational HT classification in the present study. The more negative ACT values observed in HT+ participants indicate a relative predominance of anterior temporalis over masseter activity, suggesting that the observed between-group difference primarily involved the balance of recruitment between these two jaw-elevator muscles. This interpretation is broadly consistent with previous experimental evidence showing that changes in cranio-cervical posture can selectively influence masticatory-muscle activity. Gadotti et al. reported posture-dependent changes in masticatory muscle activity during chewing, although these effects were not consistent across the masseter and anterior temporalis muscles [14]. More specifically, Xu et al. observed significant effects of head posture on both anterior temporalis and masseter activity and reported greater anterior temporalis activation in habitual or non-neutral head positions than in a neutral head position [15]. Taken together, these findings provide physiological context for a relationship between cranio-cervical posture and the relative recruitment of the jaw-elevator muscles. However, the cross-sectional nature of the present study does not allow the observed between-group differences to be interpreted as evidence of chronic neuromuscular adaptation or a compensatory recruitment mechanism.

A further noteworthy finding concerned the within-subject change in the Activity Index between the neutral head posture and anterior head flexion. ACT became significantly more negative in HT+ participants (median Δ = −4.8; r_rb = −0.637; *p* = 0.004; *q* = 0.025) and in HT− participants (median Δ = −11.1; r_rb = −0.809; *p* < 0.001; *q* = 0.002), with large within-subject effect sizes in both groups. Thus, anterior head flexion was associated with a substantial shift toward greater relative anterior temporalis recruitment irrespective of operational HT classification. Importantly, the direct between-group comparison of ΔACT was not statistically significant (*p* = 0.101; *q* = 0.607), and the corresponding effect size was small (Cliff’s δ = 0.272). Therefore, the present data do not provide sufficient evidence that the magnitude of the within-subject change in ACT between the two experimental conditions differed between HT+ and HT− participants. Moreover, because the two groups already differed substantially in ACT in the reference posture, the between-group comparison of ΔACT should be interpreted with caution. This cautious interpretation was further supported by the sensitivity analysis performed on the Activity Index, in which the Group × Posture interaction was not significant.

Previous studies investigating cervical neuromuscular behaviour provide additional context for the between-group differences observed in the present study. Experimental evidence has shown that smartphone use may influence cervical spine stability [31]. Khan et al. reported greater upper trapezius and sternocleidomastoid EMG activity both at rest and during activity in asymptomatic individuals with forward head posture compared with individuals without forward head posture [32]. Similarly, Nishikawa et al. observed altered trapezius activation characteristics in young adults with habitual forward head posture, including altered muscle activity already in the neutral position and a different posture-dependent modulation of muscle recruitment compared with participants with normal head posture [33]. Furthermore, alterations in deep cervical flexor function have been reported in young smartphone users and associated with smartphone-related exposure and forward head posture [34]. Although these studies evaluated cervical rather than masticatory muscles, they provide relevant physiological context for interpreting the between-group differences observed in the present study. However, they do not establish that the observed masticatory sEMG differences resulted from prolonged postural exposure or represent neuromuscular adaptation. This interpretation remains hypothetical, particularly because the present study was cross-sectional and cervical muscle activity was not simultaneously recorded. Longitudinal studies integrating masticatory and cervical EMG assessment are therefore needed to clarify the relationship between habitual cranio-cervical posture and these neuromuscular differences.

The present findings may also be considered in the context of previous experimental evidence indicating that changes in cranio-cervical posture can be accompanied by alterations in mandibular and condylar position. Ohmure et al. simultaneously assessed condylar position and electromyographic activity of the masticatory muscles and reported posture-related changes in both parameters, providing experimental support for a functional relationship between head posture, condylar position, and muscle recruitment [35]. In addition, in our previous three-dimensional mandibular kinematic study, Turbatu et al. documented measurable changes in condylar position and incisal relationships during anterior head flexion within a 40–60° range comparable to that used in the present study [11]. These previous findings provide a plausible biomechanical context for an interaction between cranio-cervical configuration, mandibular/condylar position, and masticatory-muscle recruitment. However, condylar position and sEMG activity were not recorded simultaneously in the present study. Therefore, the electromyographic differences observed here cannot be attributed to condylar displacement, and this potential mechanism should be regarded as hypothetical rather than directly demonstrated by the present data.

In contrast to the Activity Index, the other evaluated sEMG indices showed comparatively stable behaviour. No robust between-group or posture-related differences were identified for POC TA, POC MM, Asymmetry Index, or Torque Index after correction for multiple comparisons. Impact showed nominal reductions during anterior head flexion in both groups; however, these changes did not remain statistically significant after FDR correction (q = 0.095 in both groups). Therefore, the present findings do not provide robust evidence of a difference in overall standardized masticatory muscle activity between the two experimental conditions. Taken together, the relative stability of these indices suggests that the pattern observed across the two experimental conditions primarily concerned the relative contribution of the anterior temporalis and masseter muscles, rather than the overall muscle activity. This distinction further supports the interpretation of the observed neuromuscular pattern as selective rather than generalized.

From a clinical perspective, the present findings suggest that cranio-cervical posture may represent a relevant contextual factor when interpreting masticatory muscle sEMG indices. In particular, differences in the relative temporalis–masseter recruitment pattern should not be interpreted solely in relation to dental or occlusal factors without considering the broader cranio-cervico-mandibular functional context. An isolated sEMG pattern should not, by itself, be regarded as evidence of an occlusal cause or as an indication for irreversible modification of occlusal relationships. When clinically relevant cranio-cervical symptoms or conditions coexist, a broader multidisciplinary assessment may be appropriate, including consideration of cervical and postural factors where clinically indicated. However, the present study did not evaluate the effectiveness of physiotherapeutic, postural, or occlusal interventions. Therefore, these considerations should be regarded as implications for clinical assessment and interpretation rather than as therapeutic recommendations derived from the present findings.

Although the present study provides novel insights into posture-related neuromuscular behaviour, several limitations should be acknowledged.

Only participants with an Angle Class I dental relationship were included in the present study. This restriction was deliberately adopted to reduce occlusal and craniofacial heterogeneity and thereby limit a potential source of confounding, since sagittal craniofacial characteristics may be associated with differences in cranio-cervical posture [36,37]. However, this methodological choice also limits the generalizability of the present findings to individuals with Class II or Class III malocclusions, who may present different baseline craniofacial and biomechanical characteristics. Moreover, the classification used in the present study was dental according to Angle rather than skeletal. A more accurate characterization of sagittal skeletal relationships would have required lateral cephalometric radiography and cephalometric analysis; however, obtaining radiographic examinations solely for research screening purposes in otherwise healthy individuals without a specific clinical indication would not have been ethically justified. Therefore, the presence of underlying skeletal discrepancies not identifiable through dental classification alone cannot be completely excluded.

A major methodological limitation concerns the operational questionnaire-based classification used to define the HT+ and HT− groups. Although the illustrated questionnaire was applied using standardized images, instructions, and predefined classification criteria, it was not formally validated against objective instrumental measures of habitual cranio-cervical posture, and its test–retest reliability and reproducibility were not assessed. In particular, the operational distinction between illustrated postures 1–2 and 3–5 has not been externally validated as a threshold for distinguishing different levels of habitual postural or biomechanical exposure. Therefore, the accuracy and reproducibility of individual HT+/HT− assignment cannot be established, and some degree of group misclassification may have occurred. Moreover, because the HT− group included participants who did not simultaneously meet both criteria required for HT+ classification, it could comprise different combinations of device-use duration and habitual posture and was therefore potentially heterogeneous with respect to the two classification components. Both potential misclassification and within-group heterogeneity may have influenced the observed between-group associations. In addition, self-reported portable electronic device use duration may not accurately reflect actual daily exposure. Accordingly, the present findings should be interpreted as associations with this specific operational HT classification rather than with objectively validated categories of habitual cranio-cervical posture or biomechanical exposure. Future studies should prospectively assess the validity, test–retest reliability, and reproducibility of the HT classification against objective measurements of device-use duration and habitual cranio-cervical posture.

A further methodological limitation concerns the control of the experimental cranio-cervical position. Although anterior head flexion was standardized using predefined anatomical and postural references and an approximate target range of 40–60°, the actual flexion angle adopted by each participant was not quantitatively measured using an inclinometer, digital goniometer, motion-analysis system, or other instrumental method. Therefore, interindividual differences in the actual cranio-cervical position cannot be excluded, and it cannot be established that all participants were assessed under biomechanically equivalent experimental conditions. Such variability may have contributed to the observed sEMG responses and should be considered when interpreting the within-subject changes between the reference posture and anterior head flexion.

In addition, the order of the experimental conditions was not randomized or counterbalanced, as the reference posture was systematically recorded before anterior head flexion. Consequently, the observed within-subject changes cannot be fully separated from potential order-related effects, including familiarization with the experimental procedure, repeated MVC performance, or residual fatigue. Although a 120 s recovery interval was provided between conditions, the contribution of these potential sequence effects cannot be determined retrospectively from the available data. A further methodological limitation is that no formal intra-operator reliability analysis was performed for sEMG acquisition. Although all sEMG recordings were obtained by the same trained operator using a standardized protocol, the reproducibility of the acquisition procedure was not formally quantified. In addition, no formal reliability assessment was performed for the second observer’s verification of the experimental postural condition. Therefore, some degree of measurement and observer-related variability cannot be excluded. Reproducibility is also constrained at the signal-processing stage. Part of the processing performed by the MyoWise software relies on proprietary algorithms, and the exact procedures used for signal rectification, smoothing, and amplitude estimation were not available to the investigators. Consequently, although the same processing workflow was applied consistently across all recordings, the complete signal-processing procedure cannot be independently reproduced from the information available for the present study.

An additional limitation concerns the potential contribution of cervical symptoms to the observed sEMG differences. Self-reported neck pain was collected only as a dichotomous anamnestic variable and was not quantitatively characterized in terms of intensity, duration, frequency, associated disability, or specific clinical diagnosis using validated clinical instruments. Therefore, its potential independent contribution to the observed electromyographic differences could not be distinguished from that associated with the operational HT classification, and neck pain should consequently be regarded as a potential source of residual confounding. Other potentially relevant individual and behavioral factors, including BMI, occupation, ergonomic habits, and caffeine consumption, were also not systematically collected or included as covariates. Therefore, the observed association between the operational HT classification and the Activity Index should not be interpreted as demonstrating an independent effect of habitual device-related head posture, and residual confounding should be considered when interpreting the present findings.

The single-centre design and the relatively selected nature of the sample may also limit the external validity of the findings. In particular, the study population consisted exclusively of young, clinically healthy adults, in addition to being restricted to participants with Angle Class I dental relationships. Therefore, the present findings may not be directly generalizable to older individuals, adolescents, patients with TMDs or clinically relevant cervical or musculoskeletal conditions, or populations with different craniofacial and occlusal characteristics. In addition, the cross-sectional nature of the study precludes any inference regarding temporal or causal relationships. Accordingly, the temporal relationship between the observed electromyographic pattern and the habitual device-use and postural characteristics captured by the operational HT classification cannot be established; pre-existing recruitment characteristics or other unmeasured factors may also have contributed to the observed association.

Future studies should adopt multicentre and longitudinal designs and incorporate wearable sensors or digital monitoring systems capable of objectively recording the cumulative duration, frequency, and degree of anterior head flexion. Objective postural monitoring would reduce exposure misclassification and allow a more accurate quantification of habitual postural exposure, while longitudinal follow-up would help clarify temporal and potential dose–response relationships. In experimental protocols, cranio-cervical flexion should also be quantified using appropriate instrumental methods, and the order of postural conditions should be randomized or counterbalanced to improve standardization and minimize potential sequence effects. In addition, potential confounding factors, including cervical characteristics, neck pain, physical activity, and relevant individual and behavioural variables, should be prospectively assessed using validated quantitative measures and incorporated into appropriately specified multivariable models. This would help determine whether the observed association between the operational HT classification and masticatory sEMG outcomes persists independently of these factors. Together, these approaches may provide a more robust assessment of the relationship between portable electronic device use, cranio-cervical posture, and masticatory neuromuscular behaviour.

## 5. Conclusions

The present findings indicate that the operational HT classification was associated with differences in masticatory muscle recruitment patterns in asymptomatic individuals. Among the evaluated sEMG indices, the Activity Index showed the most consistent association with the HT classification and also showed a significant within-subject change between the reference posture and anterior head flexion. However, the magnitude of this change did not differ significantly between groups. Therefore, the present data support an association between the operational HT classification and relative temporalis–masseter recruitment, without demonstrating a differential neuromuscular response to anterior head flexion between groups. Given the cross-sectional design, no causal, temporal, or prognostic inference can be drawn from these findings.

## Figures and Tables

**Table 1:** Between-group comparison of surface electromyographic outcomes between HT+ and HT− participants.

Condition	Outcome	HT− Median [IQR]	HT− Median 95% CI	HT+ Median [IQR]	HT+ Median 95% CI	HL Median Difference HT+ − HT− [95% CI]	Cliff’s δ	*p*	*q* FDR	Analysis
Neutral head posture	POC (TA) (%)	85.8 [82.0; 87.5]	[82.1; 87.5]	86.1 [82.6; 87.5]	[83.6; 87.1]	0.2 [−1.4; 2.3]	0.042	0.808	0.889	Secondary
Anterior head flexion	POC (TA) (%)	84.2 [80.3; 87.6]	[80.5; 87.3]	85.7 [84.0; 88.0]	[85.0; 87.4]	0.8 [−1.6; 3.8]	0.133	0.426	0.889	Secondary
Neutral head posture	POC (MM) (%)	85.3 [82.4; 87.2]	[82.5; 86.8]	85.1 [81.9; 87.3]	[82.2; 87.1]	−0.1 [−2.5; 1.7]	−0.024	0.892	0.892	Secondary
Anterior head flexion	POC (MM) (%)	84.7 [80.6; 86.1]	[80.7; 86.0]	85.6 [82.2; 88.0]	[82.6; 87.5]	0.6 [−2.1; 3.2]	0.080	0.635	0.889	Secondary
Neutral head posture	ASIM (%)	4.2 [2.3; 9.5]	[2.6; 9.4]	6.0 [2.5; 10.5]	[2.6; 10.2]	0.7 [−1.5; 4.4]	0.107	0.522	0.889	Secondary
Anterior head flexion	ASIM (%)	4.8 [1.6; 10.4]	[2.0; 10.0]	6.4 [2.0; 11.9]	[2.0; 9.5]	0.3 [−2.7; 4.4]	0.045	0.793	0.889	Secondary
Neutral head posture	ACT (%)	−0.8 [−5.8; 3.3]	[−5.5; 1.4]	−17.9 [−30.3; −7.8]	[−25.0; −8.3]	−17.4 [−27.1; −9.3]	−0.688	<0.001	Not adjusted	Primary
Anterior head flexion	ACT (%)	−10.2 [−16.1; −1.7]	[−15.2; −4.1]	−18.3 [−34.2; −13.4]	[−32.4; −14.1]	−12.4 [−23.1; −4.0]	−0.493	0.003	0.032	Secondary
Neutral head posture	TOR (%)	3.7 [1.5; 6.6]	[1.6; 6.0]	5.0 [2.6; 7.3]	[3.0; 7.2]	1.0 [−0.9; 2.8]	0.144	0.388	0.889	Secondary
Anterior head flexion	TOR (%)	3.5 [2.8; 10.4]	[3.1; 7.3]	4.6 [2.8; 9.2]	[3.0; 8.3]	0.4 [−1.9; 2.9]	0.061	0.720	0.889	Secondary
Neutral head posture	IMP (%)	93.7 [79.8; 111.7]	[86.0; 106.1]	84.8 [69.8; 100.6]	[70.7; 99.8]	−10.7 [−26.7; 3.6]	−0.226	0.174	0.889	Secondary
Anterior head flexion	IMP (%)	86.7 [73.4; 100.4]	[74.2; 97.7]	84.2 [59.0; 94.6]	[65.6; 93.1]	−8.3 [−25.7; 7.2]	−0.166	0.318	0.889	Secondary

Note. Values are expressed as median [interquartile range]. Group median 95% CIs are bootstrap confidence intervals. HL median difference represents the Hodges–Lehmann estimate for HT+ minus HT− with bootstrap 95% CI. The between-group comparison of Activity Index in the neutral head posture was predefined as the primary analysis and was not adjusted for multiplicity. For secondary comparisons, *q* FDR values represent Benjamini–Hochberg false discovery rate–adjusted *p*-values within this family of comparisons. HT+ and HT−, groups defined according to the predefined operational HT classification; POC, Percentage Overlapping Coefficient; TA, anterior temporalis; MM, masseter; ASIM, Asymmetry Index; ACT, Activity Index; TOR, Torque Index; IMP, Impact; IQR, interquartile range; CI, confidence interval; HL, Hodges–Lehmann; FDR, false discovery rate.

**Table 2:** Within-group changes from reference posture to anterior head flexion in HT+ and HT− participants.

Group	Outcome	Δ Median [IQR]	Δ Median 95% CI	r_rb	*p*	*q* FDR	Analysis
HT+	POC (TA) (%)	0.2 [−1.6; 1.7]	[−1.1; 1.0]	−0.080	0.726	0.792	Secondary
HT−	POC (TA) (%)	−0.4 [−1.9; 0.2]	[−1.4; 0.1]	−0.395	0.097	0.233	Secondary
HT+	POC (MM) (%)	−0.1 [−1.9; 1.8]	[−1.3; 0.9]	−0.057	0.808	0.808	Secondary
HT−	POC (MM) (%)	−1.0 [−3.2; 1.4]	[−3.0; 1.4]	−0.293	0.209	0.358	Secondary
HT+	ASIM (%)	−0.5 [−3.3; 1.7]	[−2.4; 1.1]	−0.147	0.530	0.706	Secondary
HT−	ASIM (%)	−0.6 [−2.4; 3.1]	[−2.3; 1.6]	−0.086	0.706	0.792	Secondary
HT+	ACT (%)	−4.8 [−13.0; −1.4]	[−11.2; −1.4]	−0.637	0.004	0.025	Secondary
HT−	ACT (%)	−11.1 [−17.9; −5.9]	[−15.8; −6.6]	−0.809	<0.001	0.002	Secondary
HT+	TOR (%)	0.4 [−1.4; 1.7]	[−0.9; 1.6]	0.233	0.317	0.476	Secondary
HT−	TOR (%)	0.7 [−1.5; 4.1]	[−0.7; 4.1]	0.317	0.173	0.346	Secondary
HT+	IMP (%)	−5.2 [−9.9; 0.8]	[−9.4; 0.4]	−0.489	0.032	0.095	Secondary
HT−	IMP (%)	−6.6 [−23.1; 0.8]	[−20.1; 0.5]	−0.505	0.027	0.095	Secondary

Note. Δ was calculated as anterior head flexion minus neutral head posture. Δ median 95% CIs are bootstrap confidence intervals for the within-subject median change. *q* FDR values represent Benjamini–Hochberg false discovery rate–adjusted *p*-values within this family of secondary comparisons. HT+ and HT−, groups defined according to the predefined operational HT classification; POC, Percentage Overlapping Coefficient; TA, anterior temporalis; MM, masseter; ASIM, Asymmetry Index; ACT, Activity Index; TOR, Torque Index; IMP, Impact; IQR, interquartile range; CI, confidence interval; r_rb, matched-pairs rank-biserial correlation; FDR, false discovery rate.

**Table 3:** Between-group comparison of within-subject changes (Δ) between the neutral head posture and anterior head flexion in HT+ and HT− participants.

Outcome	HL Median Difference in Δ (HT+ − HT−) [95% CI]	Cliff’s δ	*p*	*q* FDR
POC (TA) (%)	0.5 [−0.8; 1.7]	0.154	0.357	0.823
POC (MM) (%)	0.9 [−1.3; 3.1]	0.120	0.473	0.823
ASIM (%)	−0.3 [−3.1; 2.2]	−0.046	0.786	0.823
ACT (%)	5.4 [−0.8; 11.0]	0.272	0.101	0.607
TOR (%)	−0.3 [−2.9; 1.8]	−0.038	0.823	0.823
IMP (%)	2.9 [−5.8; 12.7]	0.094	0.574	0.823

Note. Δ was calculated as anterior head flexion minus neutral head posture. Negative Cliff’s δ values indicate lower change scores in HT+ than HT−. *q* FDR values represent Benjamini–Hochberg false discovery rate–adjusted *p*-values within this family of secondary comparisons. HT+ and HT−, groups defined according to the predefined operational HT classification; POC, Percentage Overlapping Coefficient; TA, anterior temporalis; MM, masseter; ASIM, Asymmetry Index; ACT, Activity Index; TOR, Torque Index; IMP, Impact; CI, confidence interval; HL, Hodges–Lehmann; FDR, false discovery rate.

## Data Availability

Individual patients’ data are not shown for privacy reasons but are available upon reasonable request at Vita-Salute San Raffaele University, Milan, Italy.

## Supplementary Materials

The following supporting information can be downloaded at https://www.mdpi.com/article/10.3390/healthcare14182960/s1, File S1: STROBE Checklist; File S2: Posture Questionnaire.

## Abbreviations

The following abbreviations are used in this manuscript:

sEMG	Surface electromyography
TMD	Temporomandibular disorder
ICH-GCP	International Council for Harmonisation–Good Clinical Practice
STROBE	Strengthening the Reporting of Observational Studies in Epidemiology
DC/TMD	Diagnostic Criteria for Temporomandibular Disorders
FHP	Forward Head Posture
SENIAM	Surface Electromyography for the Non-Invasive Assessment of Muscles
TA	Anterior temporalis muscle
MM	Masseter muscle
POC	Percentage Overlapping Coefficient
MVC	Maximum Voluntary Contraction
ASIM	Asymmetry Index
ACT	Activity Index
TOR	Torque Index
IMP	Impact Index
IQR	Interquartile Range
R_rb	Matched-pairs rank-biserial correlation
