# Supplementary material for: Surface Electromyographic Assessment of the Cranio-Cervico-Mandibular System in Healthy Individuals with Different Head Posture Profiles During Portable Electronic Device Use
Source: Healthcare (Basel). 2026 Sep 10;14(18):2960. doi: 10.3390/healthcare14182960 (PMC13606187; doi:10.3390/healthcare14182960)
Supplement: Supplementary file 1 [file healthcare-14-02960-s001.zip › File S2 Posture Questionnaire.pdf]

POSTURE QUESTIONNAIRE

Assessment of posture, physical activity and the use of technological devices

SUPPLEMENTARY FILE
Complete English version

** Required item*

1 PERSONAL INFORMATION

1. **Surname ***
2. **Name ***
3. **Date of birth ***
4. **Sex ***
5. **Profession ***

2 POSTURAL ASSESSMENT

6. During the use of high-tech video display terminals (computers, smartphones, tablets), do you believe you maintain a correct posture? *****
   ☐ Yes ☐ No ☐ Not always

7. During the use of the device while standing, which image best represents the posture you maintain for the longest time? *****

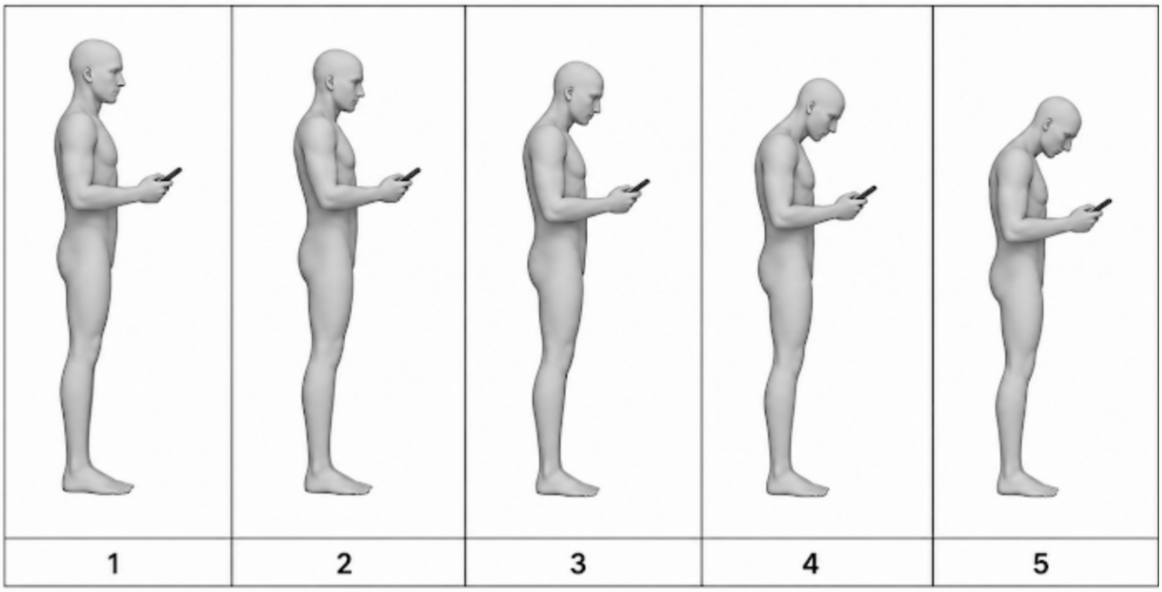

☐ 1 ☐ 2 ☐ 3 ☐ 4 ☐ 5

2 POSTURAL ASSESSMENT - CONTINUED

8. During the use of the device while seated, which image best represents the posture you maintain for the longest time? *****

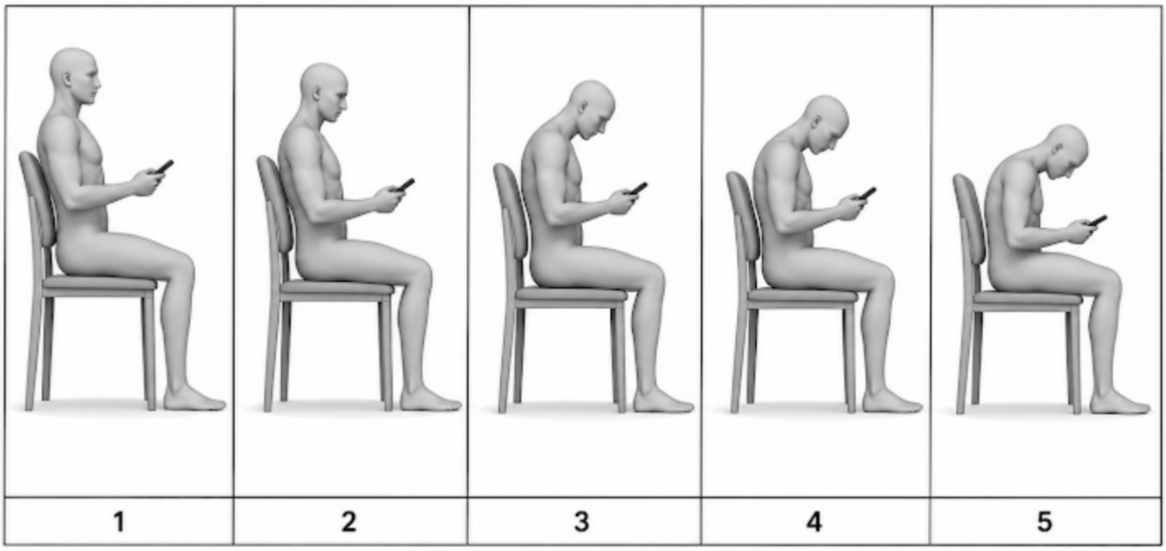

☐ 1 ☐ 2 ☐ 3 ☐ 4 ☐ 5

9. During the use of the computer, which image best represents the posture you maintain for the longest time? *****

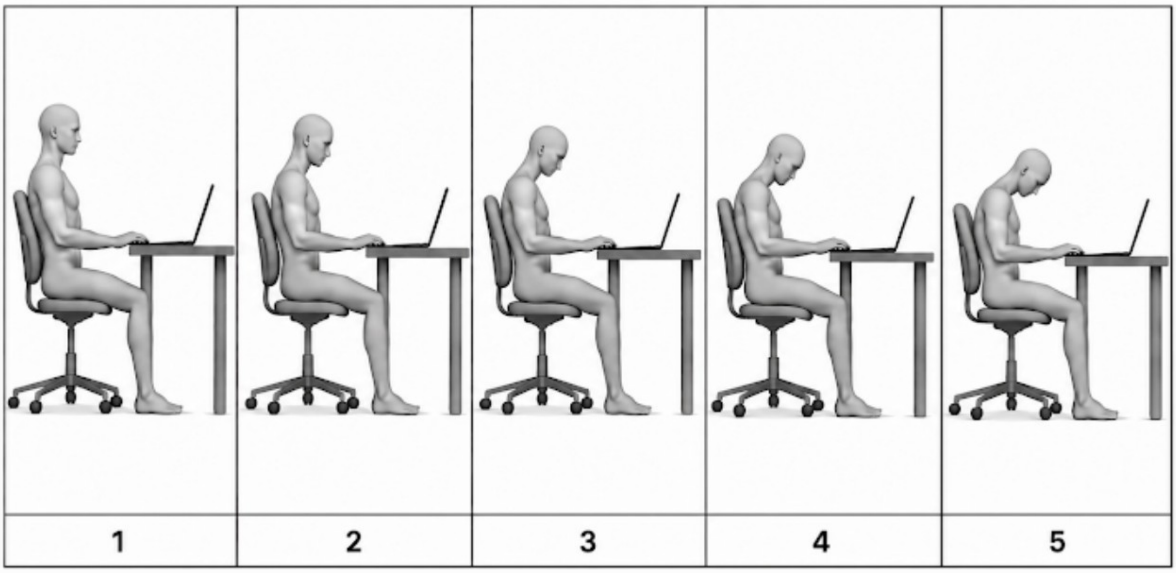

☐ 1 ☐ 2 ☐ 3 ☐ 4 ☐ 5

3 SPORTS-RELATED QUESTIONS

10. Do you practice sports / engage in physical activity? *****
☐ Yes ☐ No

11. If yes, how much time per week do you dedicate to it?
☐ 1-2 hours ☐ 2-4 hours ☐ More than 4 hours

4 USE OF HIGH-TECH DEVICES

12. Do you regularly use a portable electronic device (computer, smartphone, or tablet)? *****
☐ Yes ☐ No

13. How many hours per day do you use these devices?
☐ 0-4 h/day ☐ 5-8 h/day ☐ >8 h/day

14. Do you report episodes of cervical pain (neck pain) after using electronic devices (computer, smartphone or tablet)?
☐ Yes ☐ No

OPERATIONAL CLASSIFICATION USED IN THE STUDY

Note: This box summarizes the classification algorithm applied by the investigators and was not part of the participant-facing questionnaire.

Classification variables. HT+/HT- allocation was based exclusively on habitual cranio-cervical posture during device use (Questions 7-9) and daily device-use duration (Question 13). No total or composite questionnaire score was calculated.

HT+. Both criteria had to be simultaneously present: (1) >8 h/day of portable electronic device use (Question 13); and (2) selection of posture 3, 4, or 5 in all three postural questions (Questions 7, 8, and 9), indicating marked habitual anterior head flexion across standing, seated, and computer-use conditions.

HT-. Participants who did not simultaneously meet both HT+ criteria were classified as HT-. Thus, participants reporting 5-8 h/day did not meet the high-exposure criterion, and participants reporting >8 h/day were classified as HT- if at least one of Questions 7-9 indicated posture 1 or 2.

Variables not used for classification. Physical activity (Questions 10-11) and self-reported neck pain (Question 14) were collected only for descriptive characterization of the sample.

Instrument status and terminology. The questionnaire was developed by the research team as a study-specific operational tool. It has not undergone formal psychometric validation or specific test-retest reliability/reproducibility assessment. The >8 h/day threshold was defined a priori as an operational exposure criterion, not as a validated biological or clinical cut-off. The terms HT+ and HT- refer only to the operational postural-exposure profiles used in this study and do not denote a validated risk of TMD, neck pain, cervical disease, or other pathological conditions.

5 INFORMED CONSENT

15. Consent for the Processing of Personal Data for Research Purposes *

☐ I, the undersigned, aware of my rights regarding privacy and the processing of personal data, hereby expressly authorize the processing of my personal data in accordance with the General Data Protection Regulation (GDPR) for research purposes.

☐ I authorize the processing of my personal data for research purposes, including the collection, storage, analysis, and publication of the collected data.

☐ I confirm that I have been informed that my personal data will be processed in compliance with privacy laws and will be kept confidential and secure.

☐ I authorize the processing of my personal data for the period necessary to achieve the specified research purposes.

☐ I understand that I have the right to withdraw my consent at any time, without affecting the lawfulness of processing based on consent before its withdrawal.

I hereby give my informed consent, fully aware of its meaning and implications.

☐ Yes ☐ No

**Participant signature**	**Date**
